# Detecting Vasodilation as Potential Diagnostic Biomarker in Breast Cancer Using Deep Learning-Driven Thermomics

**DOI:** 10.3390/bios10110164

**Published:** 2020-10-31

**Authors:** Bardia Yousefi, Hamed Akbari, Xavier P.V. Maldague

**Affiliations:** 1 Department of Electrical and Computer Engineering, Laval University, Quebec City, QC G1V 0A6, Canada; 2 Department of Radiology, University of Pennsylvania, Philadelphia, PA 19104, USA; AkbariHA@upenn.edu

**Keywords:** vasodilator activity, breast cancer screening, imaging biomarker, deep sparse autoencoder, dimensionality reduction, deep-learning features

## Abstract

Breast cancer is the most common cancer in women. Early diagnosis improves outcome and survival, which is the cornerstone of breast cancer treatment. Thermography has been utilized as a complementary diagnostic technique in breast cancer detection. Artificial intelligence (AI) has the capacity to capture and analyze the entire concealed information in thermography. In this study, we propose a method to potentially detect the immunohistochemical response to breast cancer by finding thermal heterogeneous patterns in the targeted area. In this study for breast cancer screening 208 subjects participated and normal and abnormal (diagnosed by mammography or clinical diagnosis) conditions were analyzed. High-dimensional deep thermomic features were extracted from the ResNet-50 pre-trained model from low-rank thermal matrix approximation using sparse principal component analysis. Then, a sparse deep autoencoder designed and trained for such data decreases the dimensionality to 16 latent space thermomic features. A random forest model was used to classify the participants. The proposed method preserves thermal heterogeneity, which leads to successful classification between normal and abnormal subjects with an accuracy of 78.16% (73.3–81.07%). By non-invasively capturing a thermal map of the entire tumor, the proposed method can assist in screening and diagnosing this malignancy. These thermal signatures may preoperatively stratify the patients for personalized treatment planning and potentially monitor the patients during treatment.

## 1. Introduction

Breast cancers caused an estimated 41,760 deaths out of 606,808 overall deaths for females and 500 deaths for males, while the estimated new cases were 271,270 deaths for both genders (268,600 women and 2670 men) in the United States in 2019. This evidence shows that despite considerable advancement in breast cancer screening and treatment, breast cancer is still the second cause of cancer death among women [1]. Clinical breast exam (CBE), magnetic resonance imaging (MRI), mammography, and ultrasound are widely used for the diagnosis of breast cancer. Among them, CBE and mammography are considered the most common breast screening tools [1]. This study proposes machine learning techniques and analyses using infrared thermography as a new technique for breast cancer screening. We hypothesized that thermal heterogeneity may associate with angiogenesis, nitric oxide vasodilatory phenomena, inflammation, and estrogen caused by cancer symptoms.

Mammography has been the gold standard for diagnosing breast cancer since the early 1960s despite numerous studies indicating the variability of this imaging modality is affected by breast density, age, type of problem, and family history [2,3,4,5]. Mammography showed weakness in being used for breast cancer screening for women with dense breasts, hormone replacement therapy, and fibrocystic breasts [6,7,8,9]. Research showed the detection rate of mammography considerably diminishes by increasing breast density among the patients [7]. The studies show that age has a reverse association with breast cancer detection using mammography [10]. Moreover, findings show that during mammography the pressure on the breast tumors is adequate to rupture the encapsulated tumors (depends on the location of the tumor) and possibly circulate malignant cells in the bloodstream [11].

The risk of radiation using mammography is one of the important disadvantages of using this imaging modality. Younger women are more susceptible to the risk of radiation-induced breast cancer than older women due to their undifferentiated cells being prone to influence by ionizing radiation [5,10,11,12,13,14,15].

Family history of breast cancer and/or the *BRCA1/2* gene mutations are other factors that this gene mutation may be the result of radiation effect, which might be induced indirect effects to damage DNA by producing reactive oxygen species (ROS) from the cell’s water molecules [16,17]. Some studies showed that the ionizing radiation by mammography might be more dangerous for patients with such mutations [16,17,18,19].

For the dense breast, ultrasound can be an adjunctive tool used with mammography screening for detecting the abnormality [2]. However, it shows a dependency on tumor size, palpability, breast density, tools’ quality, physician’s expertise who performs the procedure, and interpreting the image [2,5,20,21,22]. Magnetic resonance imaging (MRI) is also an alternative imaging modality, which can identify early breast cancer in the place where conventional imaging fails to detect the abnormalities [23,24]. Ahen et al. (2014) concluded that the high costs and low specificity of MRI limits the popularity of MRI for annual screening for high-risk women [25]. CBE is considered as a great alternative conducted by the clinician and can help to detect at least 50% of asymptomatic breast cancers but has not been used alone [26,27].

## 2. Thermography and Biological Rationale as an Alternative Imaging Modality

Infrared thermography is used as an additional cost-effective alternative for breast cancer screening as a non-invasive procedure that does not pressurize the breast tissue nor expose the body to ionizing radiation. The skin emission is about 0.98, which is close to the emission of the blackbody. Thermal radiation emitted from the body has a wavelength of 8–10 µm bandwidth, which can be captured by the infrared camera [28,29,30]. Due to the relatively lower sensitivity of thermographic screening, it usually adds to other diagnosing methods, mainly with CBE to increase the overall diagnostic accuracy [31,32].

Blood circulation is the main contributor to heat transfer in the body. Vascularity is also considered an important parameter for heat transfer [33]. Evidence supports different thermal conductivity between normal tissues in breasts and cancerous lesion thermal profile discrepancy [34], also abnormal skin temperature manifestation is an indicator of pathological changes explained by metabolic activity associated with the tumor such as angiogenesis, nitric oxide, inflammation, and estrogen [5,33,35]. Changing in the endocrine due to the presence of tumors alters the thermal profile by changing the vascularization of the tissues to deliver oxygen and nutrients to tumors [36], in the process of pathologic angiogenesis. In such chaotic and pathological processes of angiogenesis, smooth muscle cells receiving abnormal vasoconstrict blood vessels in the area [36]. Several studies proved the value of infrared thermography on detecting hypervascularity and hyperthermia on non-palpable breast cancer [36,37,38].

Tumor angiogenesis and metastatic behavior are biomarkers of breast carcinoma and c-Met pathway activation, which are used also for tumor progression. Also, there is an association of c-Met and downstream signaling pathways with angiogenesis that can be assessed by microvessel density (MVD) [39]. Infrared was used to assess the existence of MVD, which is associated with endothelial cell (CD34) marker and downstream signaling pathways (angiogenesis, RAS-MAPK, and PI3K-AKT) [39]. In another study, there is a relationship between MVD and tumor-associated macrophages (TAMs) and vascular endothelial growth factor (VEGF) expression [23,40,41]. In addition, white blood cells produce nitric oxide as a defense mechanism against cancerous cells that is a vasodilatory substance [31,37]. Nitric oxide performs as a vasodilator in cancerous tissues in the breasts to enhance oxygen and nutrient delivery, which increases the local temperature in the area [42,43,44]. In general, cancer (including breast cancer) is influenced by different cellular factors including reactive oxygen and nitrogen species (RONS) and, as reported, hypoxic condition elevated RONS production [44,45,46,47]. Nitric oxide has reactive diatomic free radical plays a role in promoting and inhibiting cancer [48].

DNA damage occurs once nitric oxide reacts to form other reactive metabolites as well as nitrite, peroxynitrite, nitrate, or S-nitroso-thiols, which provoke genotoxic effects [48,49]. Reactive nitrogen species (RNS) exposure causes post-translational changes, which leads to different interactions by other cellular targets and causes diverse locally dependent concentration effects [49,50]. In the late stages of breast cancer also, c-Met promotes metastases by having vascular reprogramming and inflammatory cytokine upregulation [51], inflammation-related cytokine tumor necrosis factor-alpha (TNF-α) in tumor invasion [52]. This often happens due to a long period of remission before the diagnosis of breast cancer [53], which can be detected faster using the combination of CBE and infrared screening. The presence of inflammation is another mechanism of local heat generation. Cancer causes a vasodilatory response, due to the inflammatory cell involvement’ which increases temperature [42]. Estrogen also facilitates vasodilation by locally enhancing nitric oxide production. Imbalanced estrogen could change the vasodilatory effects of the tissue resulting in thermal variations [54]. Evidence shows that ecto-5′- nucleotidase (eN) is negatively controlled by estrogen receptor-α (ERα). This suggests that eN expression and its adenosine generation associate with breast cancer progression. eN expression in estrogen receptor-negative cells considered to be an aggressive breast cancer biomarker [55].

**Figure 1:**
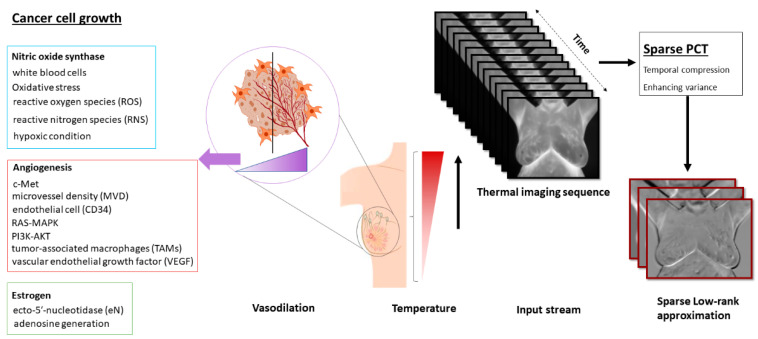
The block diagram of the biological connection to the response of infrared thermography as a fast step with other methods such as clinical breast exam (CBE) in breast cancer screening and cancer presence in the breast area are shown.

Such process metabolic heat generation investigated for normal and cancerous breast tissues and its rates reached 20K W/m^3^ and the range between 100K–1200K W/m^3^ for two types of tissues, respectively [56]. Despite United States Food and Drug Administration (FDA) approval for using infrared thermography, it can be used as an adjunct screening modality along with MRI, mammography, and ultrasound [57,58]. A visual summary of the factors influencing the heterogeneity in thermal imaging are presented in Figure 1.

In this study, we propose a method to use high-dimensional deep-learning features to track the vasodilator activities in the breast area as a potential biomarker in detecting breast cancer. The contributions of the paper are as follows:

- The sparse principal component analysis in the thermography (Sparse PCT) is used to compress the input thermal sequence and capture high temporal variance across the acquisitions. This leads to capture thermal heterogeneity patterns in three first initial bases, called *avatars*, which concatenate in three different channels similar to a red, green and blue (RGB) image as the input for our pretrained model.
- Deep thermal features, called *deep-thermomics* inspired by radiomics, are extracted to measure thermal heterogeneity in breast cancer screening using infrared thermography.
- The proposed approach tackles the problem of the *curse of dimensionality* in deep-thermomics using a sparse deep autoencoder without using traditional human-engineered feature selection methods.
- The multivariate models trained and validated using the obtained descriptors successfully classify between symptomatic and non-symptomatic subjects. We also provided a comparative analysis using a non-sparse PCT.
- This study shows the association between thermal heterogeneous patterns and potential vasodilation in the breast area, as a new potential imaging biomarker.

The rest of the paper is organized as follows. In the next section, thermal transfer in passive thermography is summarized. In Section 3, the methodology of the approach will be briefly described by applying sparse PCT analysis for thermography and pre-trained ResNet-50 deep neural networks. The experimental results are presented in Section 5, and the discussion is in Section 6. The conclusions are summarized in Section 7.

## 3. Thermal Transfer in Thermography

A thermal camera captures the spatial heterogeneity of temperature on the targeted region of interest (ROI) over time. This heat transient can be through active or passive thermography techniques. In general, the thermal transfer/heat conduction equation of a specimen can be summarized by the following equation:

(1) $$\rho C_{p}\frac{\partial T}{\partial t}=k\;\frac{\partial^{2}T}{\partial t^{2}}+\dot{q}$$

where T=T(x,y,z) is a temperature field, k is thermal conductivity constant from the material (W/m.K). ρ is the density $\left(kg/m^{3}\right)$, $C_{p}$ is specific heat (J/kg.K), $\dot{q}\left(x,y,z,t\right)$ is the internal heat generation function per unit volume, in the passive thermography.

Applying infrared thermography on biological organs and tissues, as a complex structure, composed of fat, blood vessels, parenchymal tissues, and nerves with some uncertainty for the rate of blood perfusion and metabolic activity. Pennes’ bioheat equation [59] provides accurate thermal computations and states as follows:

(2) $$\rho_{t}c_{t}\left(\frac{\partial T_{t}}{\partial t}\right)=\;\nabla .\left(k_{t}\nabla T_{t}\right)+\omega_{b}c_{b}\left(T_{a}-T_{t}\right)+q_{m}$$

where $\omega_{b}$ represents the flow rate of blood, $q_{m}$ is the metabolic rate (heat generation), and b, and a in $\omega_{b}c_{b}\left(T_{a}-T\right)$ the additive term stands for blood, and arteries (in targeted tissue), respectively.

## 4. Methodology

Infrared thermography records thermal heterogeneity in the subdermal area of the breast in temporal order. To capture such effect by abstracting such patterns, low-rank matrix approximation is used to maximize the variance across thermal acquisition time.

### 4.1. Low-Rank Approximation of Thermal Stream

Low-rank matrix approximation is commonly used in thermography [60,61,62,63,64,65,66,67,68], due to capturing thermal variations across the temporal order in the sequence. This leads to detecting thermal patterns on the subsurface of specimens. Such analyses capture thermal heterogeneity in the skin area for breast cancer screening patients. Principal component analysis (PCA), called PCT for thermography [60], through singular value decomposition (SVD) used for decomposing the input matrix (heat matrix) *X*, which is p×n, where *n* is the vectorized thermal image (breast screening) in every sequence and *p* corresponds to the number of observations and decomposes to:

(3) $$X=U\Gamma V^{T}$$

where U is the p×n matrix (p>n) and Γ is a diagonal matrix with a dimension of n×n and either zero or positive elements. $V^{T}$ denotes the transpose of the *n* × *n* matrix. The method captures the spatiotemporal variance by selecting the bases correspond to 80% of variance from the eigenvector matrix. Matrix U represents the bases of the input matrix.

The PCT is a linear transformation technique that decomposes the input zero-mean data matrix into the bases and coefficient matrix. To find the optimal solution for such transformation, $\ell_{2}$ and$\;\ell_{1}$ penalty terms with regularization parameters were added to the PCT, which led to Sparse PCT [61,62] and increased the performance of such a technique, particularly when encountering additive noise.

Such modifications in Sparse PCT not only turned PCT into a nonlinear transformation but following the same maximization of the variance among the bases. If the empirical covariance matrix of $X_{\;p\times n}$ is presented by $X^{T}X$, Sparse PCT is the maximization of variance in the direction of vector $v\in {\mathbb{R}}^{p}$ for 1≥k≥p.

(4) $$max\;v^{T}\Sigma v\;\mathrm{such}\;\mathrm{that}\;{∥v∥}_{2}=1\;,\;{∥v∥}_{0}\geq k$$

Let v be a variance of the input matrix and $∥v{∥}_{0}$ be $\ell_{0}$ norm of v, which is the non-zero components. This is an NP-hard (non-deterministic polynomial-time hard) problem and Zou et al. (2006) and elastic net algorithms used to solve this optimization [69]. Sparse PCT showed considerable performance in thermography to find a low-rank approximation of input thermal images. Here, we applied sparse PCT to preserve thermal heterogeneity in the subsurface of skin as a potential biomarker leading to early diagnosis of breast cancer.

Let I is thermal imaging stream taken from the participants such that $I\in {\mathbb{R}}^{n\times m\times \tau }$. If x is a vectorized matrix named heat matrix made by stacking vectorized infrared images, $x\;\in \;{\mathbb{R}}^{n.m\times k}$. $B=\left\{\beta_{1},\;\beta_{2},\;\ldots ,\;\beta_{\tau }\right\}$ denotes a set of bases obtained by sparse PCT. Each β cropped to a squared matrix focusing more on the ROI as an input to the ResNet-50 (spatial dimension of 224 × 224). Using k=3 corresponding to three predominant low-rank matrix approximation, we capture dynamic variations on thermal images in the ROI during τ time.

### 4.2. Deep Thermomics

Deep neural networks and particularly convolutional neural networks (CNN) are widely used by researchers in various fields with diverse applications, comprising image processing, and particularly medical imaging. CNN is a group of connected deep neural networks that uses a variation of multi-layer perceptron with many hidden layers [70]. The hidden layers of CNN normally consist of convolutional (cross-correlation) layers (filtering), pooling layers, rectifier layer (ReLu), fully connected layers, and normalization layers [71,72]. Several adaptive filters (as kernels) with small receptive fields layers makes CNN different from other similar deep neural networks. Because of such filtering in the input layers using a 2-dimensional dot product between the filter entries and the input data, the model extracts some features with higher sensitivity in spatial positions of input. This increases the applications of CNN-based networks in a variety of applications with the focus of imaging. Some of these networks are already trained for specific imaging datasets and used as a pre-trained network to perform classification or recognition.

After the success of the AlexNet [72] in image processing at the LSVRC2012, deep residual network (ResNet) [73] was perhaps the most innovative research in the computer vision and deep learning research community. ResNet provides the ability of a trainable network with many layers while holding a compelling performance.

The state-of-the-art CNN architectures are going deeper such as the very deep CNN for large-scale visual recognition (VGG) network [74], GoogleNet (also codenamed Inception-v1) [75] that have 19 and 22 layers respectively. ResNet tackle the vanishing gradient issue by introducing an “identity shortcut connection” that skips one or more layers [73], which does not degrade the network performance, since it simply stacks identity mappings in every layer. The pre-activation variant of residual block [76] increases the popularity of ResNet with an excessive number of hidden layers in the computer vision and medical image processing.

A deep learning method has been employed to non-invasively detect chemically treated collagenous tissue nonlinear anisotropic stress-strain responses in the microscopic images [77]. VGG16 is used for the prognosis of glioblastoma and as a radiographic biomarker for noninvasive categorization between true progression and pseudo-progression in these patients [78]. The initial application of the deep features in infrared analyses has been presented for finding defective patterns in the specimens using spectral difference among various areas of specimens [79]. Using traditional dimensionality reduction or feature selection is not a substantial way due to the low-level status of hidden weights in this model, which might be perceived as collinearity among features. In infrared breast cancer screening methods, a comparative analysis on AlexNet, GoogLeNet, ResNet-18, VGG-16, and VGG-19 for 88 patients using a pre-trained model resulted in discrimination between normal and pathologic patients [80]. ResNet50 was applied to extract features from histopathological images and followed by autoencoder, K-means clustering to choose discriminative patches using PCA to diagnose breast cancer [81]. A CNN approach tackled the same dataset using ResNet34 and ResNet50 and achieved a significant performance on detection of breast cancer in blind validation, and used the entire thermal sequences as the input of their system [82]. A cohort of 57 cases used applying a new configuration of CNN showed promising accuracy, while they outperformed ResNet50, SeResNet50 and Inception models [83]. Similarly, CNN used with additional algorithms such as with Bayes algorithm [84] or support vector machine (SVM) [85] to conduct diagnosis assessments.

In this paper, a pre-trained residual deep convolutional network for large-scale image recognition (ResNet-50) [73,86] was used. ResNet-50 is used as a hybrid feature generator (deep features). The method uses low-rank matrix approximation of thermal sequences as a sparse representation of the whole set, called *avatar*. Three first bases make three channels representing the entire thermal set as an input to the ResNet-50 model and extracted deep-thermomic features, 2048 size vector, as output.

**Figure 2:**
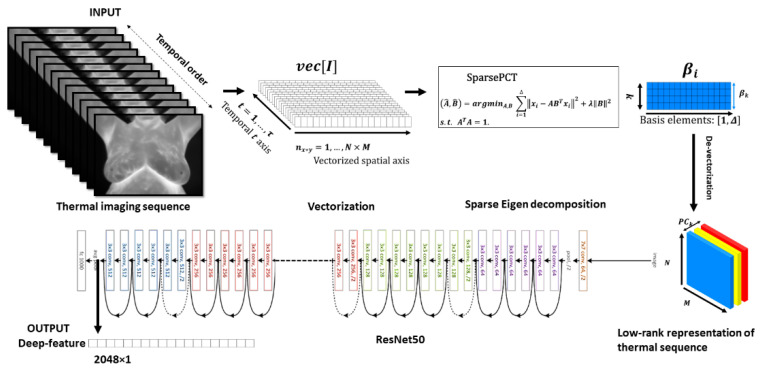
Workflow of the proposed approach in temporal compression and extraction of low-rank matrix approximation and generating the deep thermomics using residual network (ResNet-50) is presented.

Since the input of the ResNet-50 model requires an RGB squared image, we leverage this property to embed three first bases obtained by sparse PCT as three channels of the input image, showed by ψ, where $\psi_{RGB}⟶\psi_{\beta_{1}\beta_{2}\beta_{3}}$ (see Figure 2). Applying feed-forward convolution in neural network lookalikes of multiple-internal-functions gives:

(5) $$ℱ\left(\psi \right)={ℱ}_{L}\left(\ldots {ℱ}_{2}\left({ℱ}_{1}\left(\psi ;\left\{W_{1}\right\}\right);\left\{W_{2}\right\}\right)\ldots ;\left\{W_{L}\right\}\right).\;\;\;\;\;\;\;\;\;\;\;\;\;\;\;\;\;\;\;ℱ:\;{\mathbb{R}}^{224\times 224\times 3}$$

Let ${ℱ}_{i}$ represents the residual mapping to be learned and $\left\{W_{i}\right\}$ denotes weights in each layer. The regular linear convolution involves a filter bank where the output also contains the input dimensional property. The last layer contains a vector by the size of 2048 and links to three channels low-rank representation of infrared stream. This gives 2048 low-level features from the image used as input to the deep learning-based dimensionality reduction model.

### 4.3. Sparse Autoencoder for Dimensionality Reduction for Deep Thermomics

Radiomics, high-throughput features, refer to sub-visual/quantitative feature extraction and consider a vital part of medical image analysis and radiology which strive to exploit the amount of quantitative minable features extract from imaging data [87]. Every feature contains a distinct phenotype of the tumor which may have diagnosis/prognostic power and adjunctive clinical importance across the different diseases. In oncology, identified features from radiology or any other imaging data facilitate prediction, diagnosis, and prognosis associated with cancer disease to monitor the response, like survival, as a progression criterion of disease and treatment response. Pretrained deep neural networks provide high-dimensional features as an opportunity to gain information on tumor area and its environment that is not otherwise available to the radiologist.

Having sufficed features from the medical images leverage better diagnostic/prognostic decisions whereas high-dimensional feature space can impede computation and enervates the performance of feature selection known as the *curse of dimensionality* problem. This creates a wrong outcome of the model due to overfitting the decision-making unit. Traditional feature selection might not the best solution for such an issue because the low-level informative features provided by the network might translate as collinearity among the descriptors and lead to the elimination of valuable information. Here, we propose an autoencoder trained specifically for such high-dimensional throughput features to reduce the dimensionality hierarchically to the lower dimension. Autoencoders are data compression algorithms that make of hierarchical compression and decompression units cascade to each other. They are data-specific, automatically learn from training input data instead of being obtained by human interference, and lossy. Autoencoders can compress data like what they have been trained on and cannot be generalized for other dissimilar data, while they are different from lossless arithmetic compression [88]. An autoencoder contains encoding and decoding parametric functions with a measure of distance, or “loss” function, between the compressed representation of the input data and the final decompressed representation. The parameters of the encoding/decoding functions can be optimized by using stochastic gradient descent to minimize the reconstruction loss.

**Figure 3:**
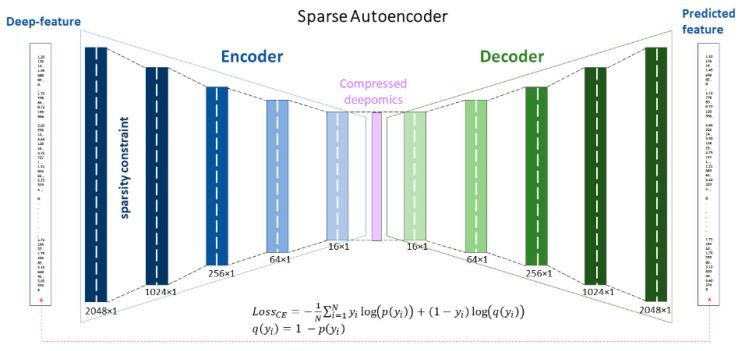
The proposed sparse deep autoencoder to reduce the dimensionality of the deep-thermomics is presented.

**Autoencoder architecture.** Several dense layers with different sizes were employed to reduce the dimensionality from 2048 to 16 compressed descriptors, in the *latent space*. Eight dense (8D) layers, including 4 dense layers in each of encoder and decoder, were used. The intermediate representation of feature dimensionality was varied from the size of 1024, 256, and then 64, to latent space with size 16. Each layer has a ReLu activation function and the last layer has a Sigmoid activation function, with sparse constraints in the initial layer (Figure 3). The network trained for the batch size of 128, with a total of 3000 iterations, with an unfixed learning rate in the Adam optimization algorithm.

Let $x\in {\mathbb{R}}^{F}$ considers as the first mapped input, where  F=2048, to the latent space with $h=f_{e}\left(x\right)=a_{e}\left(Wx+b_{e}\right)$ is the hidden representation of the input vector, $a_{e}$ is the encoder activation, $W\in {\mathbb{R}}^{F\times G}\;$ is the weight matrix, and $b_{e}\in {\mathbb{R}}^{F}$ is the encoder bias. $y=\;f_{d}\left(h\right)=a_{d}\left(W^{T}h\right)$ span the latent features back to the original space and y is the counterpart of x and $a_{d}$ is the activation for the decoder. Since we use a deep autoencoder encoder and decoder functions are expanded for multilayer as $h_{i}=f_{e_{i}}\left(\ldots f_{e_{2}}\left(f_{e_{1}}\left(x\right)\right)\right)=a_{e_{i}}\left(\ldots a_{e_{2}}\left(W_{2}a_{e_{1}}\left(W_{1}x+b_{e_{1}}\right)+b_{e_{2}}\right)\right)$ $y_{i}=\;f_{d_{1}}\left(\ldots \left(\;f_{d_{i-1}}\left(\;f_{d_{i}}\left(h_{i}\right)\right)\right)\right)=a_{d_{1}}\left(W_{1}^{T}\ldots \left(W_{i-2}^{T}a_{d_{i-1}}\left(W_{i-1}^{T}a_{d_{i}}\left(W_{i}^{T}h_{i}\right)h_{i-1}\right)\right)\right)$, respectively. The objective of an autoencoder is to minimize $\left\{W_{i},b_{e_{i}}\right\}$:

(6) $$J_{AE}=\;E_{x}\left[\ell \left(x,\;f_{d_{i}}\left(\;f_{e_{i}}\left(x\right)\right)\right)\right]$$

This captures the predominant patterns in the data and provides a noise invariant representation (manifold) of data which is very valuable considering the sensitivity of the infrared to noise. ℓ(.) denotes the loss function and here, we use binary cross-entropy (BCE), as presented below:

(7) $${ℒ}_{BCE}=\;-\;\frac{1}{F}\;\overset{F}{\underset{i=1}{\sum }}y_{i}log\left(p\left(y_{i}\right)\right)+\left(1-y_{i}\right)\log\left(1-p\left(y_{i}\right)\right)$$

where y is the label and p(y) is the predicted probability of the segmented label for all F points. Having a Sigmoid function, $\frac{1}{1+e^{-y}}$ makes the function a binarized value, representing the existing class against background class.

Learning a dictionary fitted to a training set with the sparse latent code is formulated by the optimization below [89,90]:

(8) $$min_{W_{i},\;h_{i}}\overset{F}{\underset{j=1}{\sum }}\left({∥x_{j}-W_{j}^{T}h_{j}∥}^{2}+\;\lambda {∥h_{j}∥}_{1}\right)$$

This is a convex objective in every $W_{i}$ and $h_{i}$ when the other is fixed. $\ell_{1}$ penalty term is the driving force in the above object forces for the sparse latent variable [88]. Here, the aforementioned objective is implemented for $W_{1}$ and $h_{1}$. Having sparse distributed representation (SDR) in this autoencoder not only follows the fundamental direction of deep learning but also creates robustness against noise [91]. Having our data compressed, we use a random forest to stratify the participants based on the sparse-latent deep features (Figure 3).

## 5. Results

The proposed method for thermal pattern detection was examined by thermal breast cancer screening datasets. The results of the low-rank approximation using sparse PCT were then compared to PCT thermal low-rank matrix approximation algorithm, as commonly used in infrared diagnostic systems.

### 5.1. Patient Population and Infrared Breast Cancer Study Data

We used 208 participants from Database for Mastology Research (DMR) infrared breast screening dataset [92], who were healthy (without symptoms) or sick (diagnosed by mammographic imaging as breast cancer cases or non-cancerous but with symptoms). The median age in our study sample was 60 years, and the participants comprised 77 (37%) Caucasian, 57 African (27.4%), 72 Pardo (34.6%), 1 Mulatto (0.5%), and 1 indigenous (0.5%) women. Among the participants, 52 had a history of diabetes in their families (25%), and 38 were undergoing hormone replacement (18.3%). All patients had infrared images obtained by the following acquisition protocol: images have a spatial resolution of 640 × 480 pixels and were captured by a FLIR thermal camera (model SC620) with a sensitivity of less than 0.04 °C range and capture standard of −40 °C to 500 °C [27,92]. Table 1 shows the clinical information and demography of the cohort. In this study, we considered symptomatic patients (who are not diagnosed with cancer but have similar signs) and sick (cancer) patients in one group, called the symptomatic group. The rationale behind this is due to having such analyses as the first line of screening, and once heterogeneity is detected by this system, further investigations need to be performed by a physician and another imaging modalities, i.e., mammography, to confirm the malignancy and specify the possible type of tumor.

### 5.2. Results of Low-Rank Sparse PCT (Principal Component Analysis)

**Figure 4:**
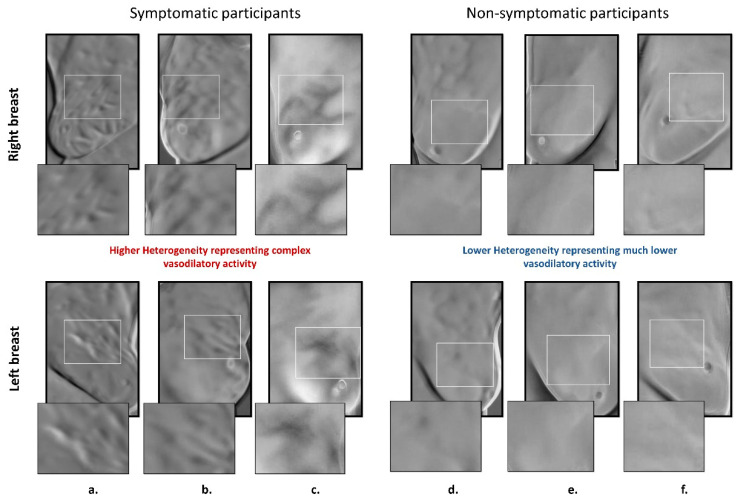
Low-rank approximation of thermal sequence determined using different Sparse PCT (principal component analysis) matrix factorization technique. Each column shows different case, columns (**a**–**c**) show symptomatic patients (diagnosed by mammography as cancer patients or healthy with symptoms), whereas columns (**d**–**f**) show the result of methods for healthy cases.

Three low-rank matrices were extracted from the 23 initially thermal sequences by using Sparse PCT. Some representative results of the low-rank approximation manually selected for our study cohort are shown in Figure 4. Low-rank approximation in the sequence of thermal images resulted in a heterogeneous breast area for 80 participants for breast cancer screening (sick and healthy with symptoms versus completely healthy without any symptom, Figure 4a–c). Thermal patterns showed more heterogeneous textures presenting the vasodilatory effect on the subdermal area of the breast. However, there was much less thermal heterogeneity found among the healthy participants (Table 1, Figure 4d–f). The targeted areas indicate significantly lesser heterogeneous patterns projected by the low-rank Sparse PCA in the ROI.

### 5.3. Deep-Thermomic Features

We extracted 2048 deep thermomic features from the targeted ROI in the thermal imaging (solely breasts area) using the latest layer of the ResNet-50 pre-trained model. The ResNet-50 pre-trained model contained five identical blocks having a convolutional layer, max-pooling, ReLu, and many repetitive identity connections between each layer. Convolutional blocks consisted of three convolution layers like identity block. The ResNet-50 model had 25,583,592 trainable parameters and 53,120 non-trainable parameters. The preferred input image to ResNet-50 was an RGB image with a dimension of 224×224×3. This squared image slipped through the entire process and ResNet-50 model re-scaled the spatial dimensions of the input image from 224 to 230, 112, 56, 28, 14, 7 while the fourth dimension grew from 3 to 64, 128, 256, 512, and 2048. Here, we used this to leverage the low-rank matrix approximation for each participant. We extracted three bases using sparse low-rank matrix approximations from the original thermal stream and stacked them like three channels in the input image. The input image was cropped around the ROI to create a square matrix for each channel identically.

### 5.4. Result of the Sparse Autoencoder and Dimensionality Reduction

**Figure 5:**
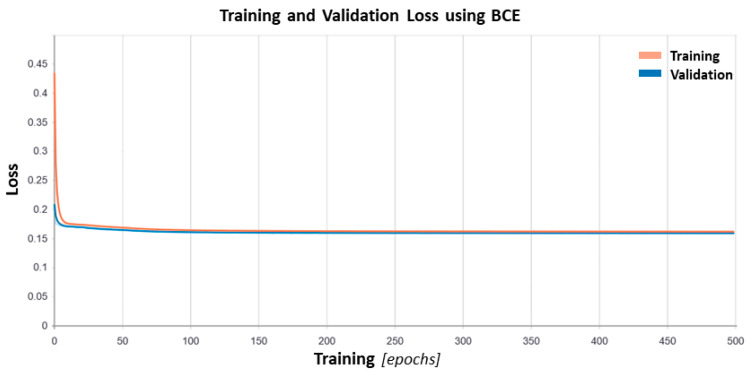
The binary cross entropy loss is presented for training and validation of the proposed sparse autoencoder for 300 epochs.

We used 2048 deep features extracted from the ResNet-50 pre-trained model as the input of the proposed autoencoder. The autoencoder consisted of seven layers (Figure 3) and reduced the dimensionality from 2048 to 1024, 256, 64, and 16. The model was trained and validated with 4000, and 2000 vectors obtained by the ResNet-50 model from infrared images in the breast screening dataset. The model had 4,744,784 trainable parameters and was trained by *Adam* optimizer with the learning $\ell_{1}$ with a regularization value of 10^−5^ and for 500 epochs. The batch size was 128 for the model network. Figure 5 shows the loss of the model during the training.

From 2048 initially extracted deep thermomic features, the extracted features from the battle-neck layer of autoencoder resulted in 16 deep-thermomic features (Figure 5). Subsequently, the level of the heterogeneity for each participant measured through compressed descriptors was obtained by 128 times compression on the deep-thermomics.

### 5.5. Result of Random Forest Classification of Symptomatic and Non-Symptomatic Participants

**Figure 6:**
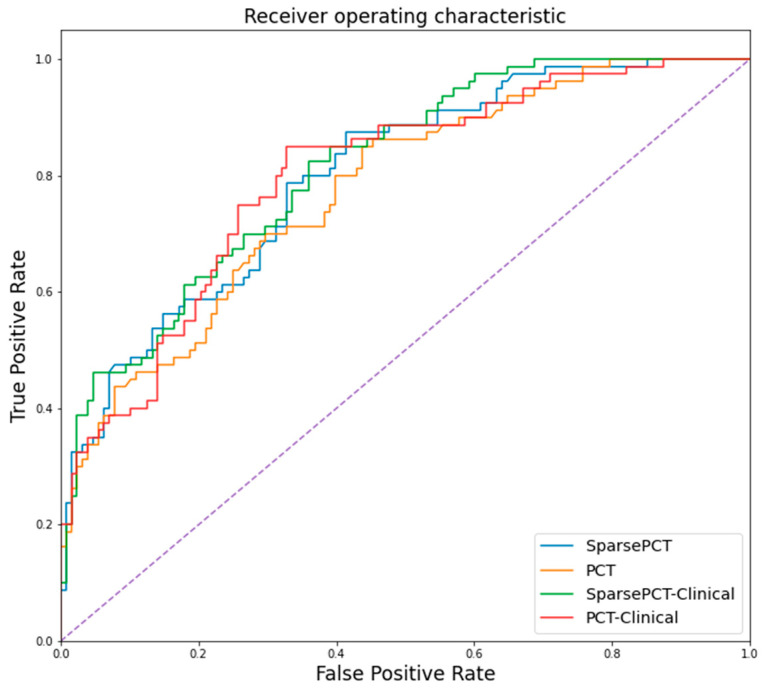
Receiver operating characteristic (ROC) curve for different multivariate model using deep thermomic features and clinical and demographic information is presented for classifying between symptomatic and non-symptomatic participants (for baseline model).

We stratified the participants based on the 16 sparse latent deep thermomic descriptors and compared them with the ground truth data based on mammography information. To examine the hypothesis that the thermal heterogeneity extracted by the deep learning model can be used as a biomarker to stratify among participants, a random forest classifier was fitted for multivariate covariates with leave-one-out cross-validation. The best multivariate model resulted accuracy of 75.24% (72.33–77.67%) for Sparse PCT, which was challenged by other matrix approximation technique PCT (73.27% (71.84–76.21%)). A multivariate model contains clinical information and demographics (age, and family history) gave an accuracy of 71.36% (69.42–73.3%). A full multivariate model having all clinical and demographic information with the extracted features resulted in 78.16% (73.3–81.07%) for Sparse PCT and 73.79% (72.33–76.7%) for PCT (see Table 2). The receiver operating characteristic (ROC) curve of comparative analyses of baseline models is shown in Figure 6. The entire computational experiments were conducted by Python programming language [93] (for training and testing the model).

## 6. Discussion

In this study, we proposed a system to reduce the dimensionality of deep-thermomic features to extract thermal patterns for infrared diagnostic systems for thermography imaging. This study was designed based on the general trend of dimensionality reduction and to alleviate the possibility of over-fitting but used sparse multiple low-rank matrix approximations. This study showed a possibility to identify potential patients with breast cancer along with other clinical throughputs (such as CBE) using non-invasive, faster, and more cost-efficient thermography imaging.

The application of deep sparse autoencoder not only reduced the initial high-dimensional deep-thermomics but also added sparsity to the initial sparse representative of the thermal stream, which theoretically increases the robustness of the system against noise. It also showed significant improvements in stratifying symptomatic patients from healthy participants (Figure 6, and Table 2). Moreover, using sparse PCT showed higher accuracy than other approaches in finding heterogeneous thermal patterns, which might be due to the nature of sparsity in the calculation of the low-rank representative of the basis matrices, which were preserved by ResNet-50 level-level features and recursive training of the autoencoder network. This indicates the penalty terms in Sparse PCT creates constraints that worked in favor of detecting symptomatic cases while eliminating noise.

The application of deep thermomics considerably increased the dimensionality of the input thermal imaging and intensify the possibility of overfitting the random forest model, called the *curse of dimensionality*. The proposed sparse autoencoder reduced the dimensionality by removing the redundancy among the features by spanning thermomics to lower-dimensional space, while increasing the robustness of feature selection due to rigorous training of the model (this method is also used in other applications in medicine such as segmentation [94]). Since the infrared images used in this study had intensity information similar to natural images, using this pre-trained model is seemingly appropriate despite the medical nature of the analysis.

Thermal and infrared imagery has been used to determine breast abnormality, as the first medical application of thermography [92]. There are many discussions about more suitable positions for such imaging acquisitions [35] and the reliability of this modality [57,58] that have been reported. However, the association of sparse autoencoder on the abundant deep thermomics finding thermal heterogeneity with a breast abnormality has not been discussed in literature which increases the novelty of this contribution to the field. One of the reasons that the proposed system performs well finding thermal heterogeneity might be because of the association of low-level deep features representing basis set and their sensitivity to slight intensity variation of thermal images. This can be justified by vasodilatory activity in the ROI for symptomatic patients. Despite some argument against using a thermal infrared imaging system as a solo-imaging modality for detecting breast abnormality, this technology has still been used as one of the important diagnostic tools with CBE and other imaging modalities (as discussed in Section 2).

One limitation to applying the presented models is related to data, and even with a considerable number of cases there is a need to increase the cohort to confirm the accuracy of the system. Having a larger cohort of patients increases the statistical power of such analysis by increasing the possibility of independently validating the system (substitute cross-validation). The other limitation may be using limited deep thermomic features. Having more deep-thermomics helps to assess the strength of the sparse autoencoder approach to select better compressing features that lead to capturing better thermal characteristics. There is an inherent limitation of using infrared thermography for detecting cancerous tissues once they are deeper into the tissues, as despite tracking the vasodilatory effects in the skin subsurface, deeper lesions might not be easy to detect. This might require further investigation using multimodal imaging analyses. There are also discussions on using a single thermal frame or multiple frames for selecting variability of thermal patterns across the acquisition time using different infrared-based approaches [5,35,95,96,97], which can be investigated further. Applying multiple thermal frames provided a chance of capturing thermal heterogeneity in the ROI for the duration of the acquisition, which might not be recorded by a single frame input system.

The presented technique offers some advantages. First, applying low-rank matrix approximation to extract thermal *avatars* during imaging acquisition provides a significant projection of thermal heterogeneity leading to better diagnosis of abnormal patients. Second, a sparse autoencoder eliminates the manual selection or human-engineering feature selection for reducing the dimensionality of the deep thermomics. Third, the proposed method considerably alleviates the effect of motion artifacts and imaging acquisition noise, which can be substantial improvements in infrared thermography applications. To the best of our knowledge, this is the first study which performs such analyses.

## 7. Conclusions

This study addressed one of the biggest challenges in high-dimensional deep feature selection, which selected the best representative deep thermomics from high-dimensional features extracted from a pre-trained deep neural networks model. The method performed multilayer dimensionality reduction using Sparse PCT to select the low-rank approximation of the thermal sequence. They extracted high-dimensional features from the ResNet-50 pre-trained model. Then, it used a trained sparse autoencoder to hierarchically reduce the size of the feature to 16 thermomic descriptors. We tested our method for 208 thermal breast cancer screening cases. We compared the appropriateness of these approaches with similar state-of-the-art thermographic methods, i.e., PCT. The results indicated the significant performance of the full multivariate model using Sparse PCT in preserving thermal heterogeneity to discriminate between symptomatic and healthy participants (accuracy of 78.16% (73.3–81.07%)).

Future works should involve more thermomics extracted from the different low-rank approximations to increase the potential of assessing the entire thermal characteristics of cancerous parenchymal tissues. Moreover, an expansion of the validation set to a larger infrared imaging cohort can further confirm the strength and limitations of this approach.

## Figures and Tables

**Table 1:** Clinical information and demographics of the breast cancer screening database using thermal imaging.

DMR—Database for Mastology Research		
**Age**	Median (±IQR)	60 (25,120)
**Race**	Caucasian	77 (37%)
	African	57 (27.4%)
	Pardo	72 (34.6%)
	Mulatto	1 (0.5%)
	Indigenous	1 (0.5%)
**Diagnosis ^1^**	Healthy ^2^	128 (61.5%)
	Symptomatic (with and without cancer)	80 (38.5%)
	Sick ^3^	36 (17.3%)
**Family history**	Diabetes	52 (25%)
	Hypertensive	5 (2.4%)
	Leukemia	1 (0.5%)
	None	150 (72.1%)
**Hormone therapy (HT)**	Hormone replacement	38 (18.3%)
	None	170 (81.7%)

^1^ This diagnosis performed with mammography as ground truth in this Dataset. ^2^ Healthy term is used as non-cancerous and non-symptomatic patients. ^3^ We use the term “sick”, which includes different types of breast cancer patients diagnosed by mammographic imaging.

**Table 2:** The results of random forest classification for the cross-validated model.

Methods	Cross-Validated Accuracy
**Sparse PCT**	75.24 (72.33–77.67)%
**PCT**	73.27 (71.84–76.21)%
Clinical information *	71.36 (69.42–73.3)%
Sparse PCT with clinical information *	78.16 (73.3–81.07)%
PCT with clinical information *	73.79 (72.33–76.7)%

* Clinical and demographic covariates: age, and family history.
